# Impact of compensated cirrhosis on survival in patients with acute-on-chronic liver failure

**DOI:** 10.1007/s12072-021-10266-8

**Published:** 2021-11-25

**Authors:** Kessarin Thanapirom, Tongluk Teerasarntipan, Sombat Treeprasertsuk, Ashok Choudhury, Manoj K. Sahu, Rakhi Maiwall, Viniyendra Pamecha, Richard Moreau, Mamun Al Mahtab, Yogesh Kumar Chawla, Harshad Devarbhavi, Chen Yu, Qin Ning, Deepak Amarapurkar, Chundamannil E. Eapen, Saeed Sadiq Hamid, Amna Subhan Butt, Dong Joon Kim, Guan H. Lee, Ajit Sood, Laurentious A. Lesmana, Zaigham Abbas, Gamal Shiha, Diana A. Payawal, Man-Fung Yuen, Albert Chan, George Lau, Jidong Jia, Salimur Rahman, Barjesh C. Sharma, Osamu Yokosuka, Shiv Kumar Sarin, The APASL ACLF Working Party, Shiv Kumar Sarin, Ashok Choudhury, Manoj K. Sharma, Rakhi Maiwall, Mamun Al Mahtab, Salimur Rahman, Sanjiv Saigal, Neeraj Saraf, A. S. Soin, Harshad Devarbhavi, Dong Joon Kim, R. K. Dhiman, Ajay Duseja, Sunil Taneja, C. E. Eapen, Ashish Goel, Q. Ning, Tao Chen, Ke Ma, Z. Duan, Chen Yu, Sombat Treeprasertsuk, S. S. Hamid, Amna S. Butt, Wasim Jafri, Akash Shukla, Vivek Saraswat, Soek Siam Tan, Ajit Sood, Vandana Midha, Omesh Goyal, Hasmik Ghazinyan, Anil Arora, Jinhua Hu, Manoj Sahu, P. N. Rao, Guan H. Lee, Seng G. Lim, Laurentius A. Lesmana, Cosmas Rinaldi Lesmana, Samir Shah, V. G. Mohan Prasad, Diana A. Payawal, Zaigham Abbas, A. Kadir Dokmeci, Jose D. Sollano, Gian Carpio, Ananta Shresta, G. K. Lau, Md. Fazal Karim, Gamal Shiha, Rino Gani, Kemal Fariz Fariz Kalista, Man-Fung Yuen, Seema Alam, Rajeev Khanna, Vikrant Sood, Bikrant Bihari Lal, Viniyendra Pamecha, Ankur Jindal, V. Rajan, Vinod Arora, Osamu Yokosuka, Madunil A. Niriella, Hai Li, Xiaolong Qi, Atsushi Tanaka, Satoshi Mochida, Dominic Ray Chaudhuri, Ed Gane, Khin Maung Win, Wei Ting Chen, Mohd Rela, Dharmesh Kapoor, Amit Rastogi, Pratibha Kale, Archana Rastogi, Chhagan Bihari Sharma, Meenu Bajpai, Virender Singh, Madhumita Premkumar, Sudhir Sudhir, A. Olithselvan, Cyriac Abby Philips, Anshu Srivastava, Surender K. Yachha, Zeeshan Ahmad Wani, B. R. Thapa, Anoop Saraya, Shalimar, Ashish Kumar, Manav Wadhawan, Subash Gupta, Kaushal Madan, Puja Sakhuja, Vivek Vij, Barjesh C. Sharma, Hitendra Garg, Vishal Garg, Chetan Kalal, Lovkesh Anand, Tanmay Vyas, Rajan P. Mathur, Guresh Kumar, Priyanka Jain, Samba Siva Rao Pasupuleti, Yogesh K. Chawla, Abhijit Chowdhury, Shahinul Alam, Do Seon Song, Jin Mo Yang

**Affiliations:** 1 Division of Gastroenterology, Department of Medicine, Faculty of Medicine, Thai Red Cross, King Chulalongkorn Memorial Hospital, Chulalongkorn University, Pathumwan, Bangkok, Thailand; 2 Liver Fibrosis and Cirrhosis Research Unit, Chulalongkorn University, Bangkok, Thailand; 3 Department of Hepatology, Institute of Liver and Biliary Sciences, New Delhi, India; 4 IMS &SUM Hospital, Bhubaneswar, Odisha India; 5 Department of Hepatology, Institute of Liver and Biliary Sciences, D-1, Vasant Kunj, New Delhi, India; 6 Department of Liver Transplantation and Hepato Pancreatico Biliary Surgery, Institute of Liver and Biliary Sciences, New Delhi, India; 7 EF Clif, EASL-CLIF Consortium and Grifols Chair, Barcelona, Spain; 8 Inserm, U1149, Centre de Recherche Sur L’Inflammation (CRI),, Paris, France; 9 UMRS1149, Université de Paris, Paris, France; 10 Service d’Hépatologie, Hôpital Beaujon, Assistance Publique-Hôpitaux de Paris, Clichy, France; 11 Bangabandhu Sheikh Mujib Medical University, Dhaka, Bangladesh; 12 PGIMER, Chandigarh, India; 13 St John’s Medical College, Bangalore, India; 14 Beijing Youan Hospital and Translational Hepatology Institute, Beijing, China; 15 Tongji Hospital, Tongji Medical College, Wuhan, China; 16 Department of Gastroenterology, Bombay Hospital and Medical Research Centre, Mumbai, India; 17 CMC, Vellore, India; 18 Aga Khan University Hospital, Karachi, Pakistan; 19 Hallym University College of Medicine, Chuncheon, South Korea; 20 Yong Loo Lin School of Medicine, National University of Singapore, Singapore, Singapore; 21 Dayanand Medical College, Ludhiana, India; 22 Digestive Disease & Oncology Centers, Medistra Hospital, Jakarta, Indonesia; 23 Ziauddin University, Karachi, Pakistan; 24 Egyptian Liver Research Institute and Hospital, Cairo, Egypt; 25 Cardinal Santos Medical Center, San Jaun, Philippines; 26 Queen Mary Hospital, The University of Hong Kong, Pok Fu Lam, Hong Kong; 27 Department of Medicine, Humanity and Health Medical Group, New Kowloon, Hongkong, China; 28 Friendship Hospital, Capital University, Beijing, China; 29 Department of Hepatology, Institute of Liver and Biliary Sciences, D-1, Acharya Shree Tulsi Marg, Vasant Kunj, New Delhi, 110070 India; 30 Department of Advanced Endoscopy, Institute of Liver and Biliary Sciences, D-1, Acharya Shree Tulsi Marg, Vasant Kunj, New Delhi, 110070 India; 31 Chiba University, Chiba, Japan; 32 Institute of Liver and Biliary Sciences, New Delhi, India

**Keywords:** Cirrhosis, Acute-on-chronic liver failure, Mortality, Prognosis, Chronic liver disease, Liver injury

## Abstract

**Background and aims:**

Acute-on-chronic liver failure (ACLF) is considered a main prognostic event in patients with chronic liver disease (CLD). We analyzed the 28-day and 90-day mortality in ACLF patients with or without underlying cirrhosis enrolled in the ACLF Research Consortium (AARC) database.

**Methods:**

A total of 1,621 patients were prospectively enrolled and 637 (39.3%) of these patients had cirrhosis. Baseline characteristics, complications and mortality were compared between patients with and without cirrhosis.

**Results:**

Alcohol consumption was more common in cirrhosis than non-cirrhosis (66.4% vs. 44.2%, *p* < 0.0001), while non-alcoholic fatty liver disease/cryptogenic CLD (10.9% vs 5.8%, *p* < 0.0001) and chronic HBV reactivation (18.8% vs 11.8%, *p* < 0.0001) were more common in non-cirrhosis. Only 0.8% of patients underwent liver transplantation. Overall, 28-day and 90-day mortality rates were 39.3% and 49.9%, respectively. Patients with cirrhosis had a greater chance of survival compared to those without cirrhosis both at 28-day (HR = 0.48; 95% CI 0.36–0.63, *p* < 0.0001) and 90-day (HR = 0.56; 95% CI 0.43–0.72, *p* < 0.0001), respectively. In alcohol CLD, non-cirrhosis patients had a higher 28-day (49.9% vs. 23.6%, *p* < 0.001) and 90-day (58.4% vs. 35.2%, *p* < 0.001) mortality rate than cirrhosis patients. ACLF patients with cirrhosis had longer mean survival than non-cirrhosis patients (25.5 vs. 18.8 days at 28-day and 65.2 vs. 41.2 days at 90-day). Exaggerated systemic inflammation might be the reason why non-cirrhosis patients had a poorer prognosis than those with cirrhosis after ACLF had occurred.

**Conclusions:**

The 28-day and 90-day mortality rates of ACLF patients without cirrhosis were significantly higher than those with cirrhosis in alcoholic CLD. The presence of cirrhosis and its stage should be evaluated at baseline to guide for management. Thai Clinical Trials Registry, TCTR20191226002.

**Supplementary Information:**

The online version contains supplementary material available at 10.1007/s12072-021-10266-8.

## Introduction

Acute-on-chronic liver failure (ACLF) is a distinct syndrome that is described in patients with chronic liver disease (CLD) who have acute deterioration of liver function after precipitated by certain factors and results in severe hepatic dysfunction with or without extra-hepatic organ failure, which leads to high short-term mortality [1]. The prevalence of ACLF is increasing and varies between 23 and 74% in hospitalized cirrhotic patients [1]. ACLF consumes numerous healthcare resources and poses a financial burden with unsatisfactory treatment outcomes [7–9]. Ideally, intensive management and monitoring should be given for all ACLF patients; nonetheless, many factors in the real world limit us from providing intensive care to every patient. Patient selection and allocation of appropriate treatments according to the disease severity and prognosis are some of the most effective strategies to achieve good treatment outcomes, especially in situations of limited resources. Therefore, to select suitable patients, adequate prognostic predictors are essential.

A number of literatures have tried to identify factors that could be utilized as prognostic predictors. Unfortunately, results from various studies are inconsistent and cannot be concluded. The CLIF-SOFA score, based on the types and number of organ failure, is regarded as the best tool for diagnostic accuracy to predict ACLF mortality [2, 3]. However, this score was developed on the basis of clinical presentation and complications after ACLF has occurred. In contrast, the pre-ACLF status of liver histology and function are not included in the consideration. An original study enrolling patients was carried out with cirrhosis cases only, but it was later extrapolated to apply with any CLD patients, which shows that the effects of cirrhosis on the prognosis ACLF has not been well studied.

In the initial ACLF definitions, European Association for the Study of the Liver (EASL) and the American Association for the Study of Liver Disease (AASLD), emphasized only patients with cirrhosis. In contrast, the Asia Pacific Association for the Study of the Liver (APASL) definition included both cirrhosis and non-cirrhosis patients. The APASL ACLF Research Consortium (AARC) data demonstrated that non-cirrhotic CLD patients with ACLF have a high 4-week mortality rate (above 33%) [4]. Taking this into account, the World Gastroenterology Organization (WGO) consensus included patients with or without cirrhosis in their definition of CLD underlying ACLF [5]. There are several evidences which show that even in the absence of cirrhosis, CLD patients who develop ACLF have a poor prognosis. In fact, even in the subsequent reports from the CANONIC study, the patients with cirrhosis were reported to be doing better than the non-cirrhosis. There is, however, limited information on the influence of the baseline degree of portal hypertension and CLD on the severity and outcome of the patients who have suffered acute decompensation. There are also very few studies that have primarily evaluated the presence or absence of cirrhosis on the outcome of patients with ACLF. The present study was undertaken to analyze the prospectively collected data from the AARC database to assess the 28-day and 90-day mortality rate of ACLF patients with respect to the presence of underlying cirrhosis.

## Patients and methods

### Study design

On behalf of the APASL ACLF Research Consortium (AARC) (detailed information is made available at http://www.aclf.in/), we prospectively collected information from the hospitalized patients. The study period was from October 2009 to April 2016. All adult patients age above 18 years who were hospitalized from the emergency room or outpatient clinical due to having ACLF, were included in the study. Liver cirrhosis was diagnosed based on the radiological imaging findings of liver nodularity and/or portal hypertension. Histopathological study was performed in selected cases. A diagnosis of ACLF was defined according to the APASL criteria: jaundice (serum bilirubin ≥ 5 mg/dl) and coagulopathy (INR ≥ 1.5 or prothrombin activity < 40%), followed by ascites and/or encephalopathy within 4 weeks in patients with prior diagnosed or undiagnosed chronic liver disease or cirrhosis [4, 6]. Patients with any of the following; decompensated cirrhosis, concomitant malignancy, pregnancy, severe comorbidities (such as severe neurological deficit, poor cardiac function, chronic lung disease that limits activity in daily life, uncontrolled autoimmune diseases, etc.), chronic kidney disease, and currently received immunosuppressant, were excluded from the study.

Patients with ACLF were investigated to determine the precipitating cause, etiology of liver disease, and severity of hepatic decompensation at the time of presentation. Standard medical care and management of organ dysfunction and failure were provided to every patient. The course of the patients and the final outcome were analyzed till 90 days. At the end of the study, all parameters were compared between groups of ACLF with and without cirrhosis. Statistical analyses were performed by authors who were not involved in data collection or treatment.

This study was reviewed and approved by the Institutional Review Board of the Faculty of Medicine, Chulalongkorn University (IRB No. 330/59). This study was registered in the Thai Clinical Trials Registry, ID TCTR20191226002. All authors had access to the study data, and they reviewed and approved the final manuscript.

### Statistical analysis

Categorical variables were described as counts and percentages and were compared using Fisher's exact test. Continuous variables were demonstrated as means and standard deviations. The independent sample t-test and Mann–Whitney (Wilcoxon rank) test were used to compare groups with and without normal distribution. Primary and secondary outcomes were 28-day and 90-day mortality rates. Kaplan–Meier method was used in survival analysis. The log-rank (Mantel-Cox) test was used to evaluate survival of cirrhosis and non-cirrhosis groups, which followed a Chi-square distribution. Crude analysis was demonstrated by the Kaplan–Meier curves and compared by the mean survival time between each group. Multivariate analysis by Cox regression was performed to assess the relationship between potential factors that might affect mortality. The parameters in the multivariate analyses were considered by two types according to the onset of ACLF development; (1) pre-ACLF parameters including age, sex, CLD etiologies and precipitating factors, and (2) post-ACLF parameters including the initial laboratory profiles, CTP and MELD scores, the presence of hepatic encephalopathy, ascites and number of organ failure. The Hazard ratio was used to estimate the direction and degree of each variable to the prognosis of ACLF at the 28-day and 90-day periods.

## Results

**Fig. 1:**
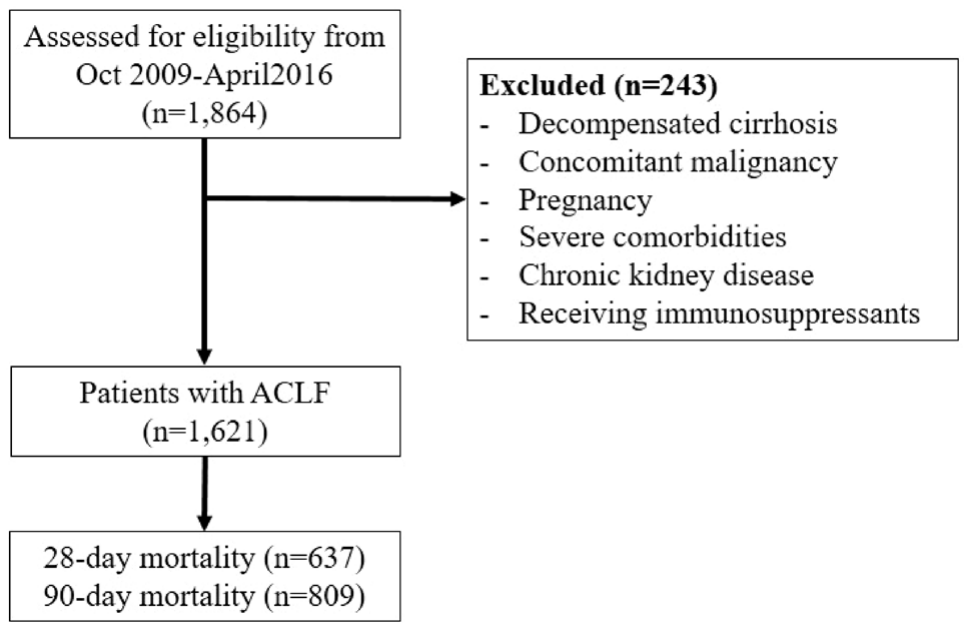
The Strengthening the Reporting of Observational Studies in Epidemiology (STROBE) flowchart of patient enrolment

Strengthening the Reporting of Observational Studies in Epidemiology (STROBE) flowchart of patient enrolment is shown in Fig. 1. A total of 1621 hospitalized ACLF patients were enrolled in the study; 1405 of them were male (86.7%) with a mean age of 44.6 ± 11.8 years. Alcohol consumption, followed by hepatitis B virus (HBV) infection and reactivation, seemed to be a major health issue because prevalence was by far more common than other factors in terms of both underlying and precipitating factors. HBV, hepatitis E virus (HEV) reactivation, and drug-induced liver injury as causes of liver decompensation were frequently found in our study, while these were considered uncommon in western countries. The mean baseline MELD and CTP scores were 29 ± 7 and 12 ± 1.5, respectively. Most patients (88.8%) had one or two major organ dysfunctions on admission. The most frequent organ failures were liver (79.8%), coagulation (35.3%), and renal (23.2%). The average length of hospital stay was 17.5 ± 14.1 days. The overall 28-day and 90-day mortality rate were 39.3% and 49.9%, respectively (Table 1). The average age in the non-cirrhosis CLD group was statistically higher than cirrhosis CLD group, but was not significant. The proportion of males was higher in the cirrhosis patients than the non-cirrhosis patients.

**Table 1:** Baseline characteristics of patients with acute-on-chronic liver failure

Characteristics	Overall (*n* = 1,621)	Cirrhosis (*n* = 637)	Non-cirrhosis (*n* = 984)	*p*-value
Age (years)	44.0 ± 11.9	44.0 ± 10.7	45.0 ± 12.5	0.040
Male, *n* (%)	1,405 (86.7%)	576 (90.4%)	829 (84.2%)	< 0.0001
CLD etiologies	*N* = 1460	*N* = 631	*N* = 829	
Alcohol	858 (58.8%)	423 (67.0%)	435 (52.5%)	< 0.0001
HBV	274 (18.8%)	98 (15.5%)	176 (21.2%)	0.007
HCV	42 (2.9%)	10 (1.6%)	32 (3.9%)	0.011
NASH	81 (5.5%)	37 (5.9%)	44 (5.3%)	0.646
Cryptogenic	144 (9.9%)	37 (5.9%)	107 (12.9%)	< 0.0001
Other causes^a^	61 (4.2%)	26 (4.1%)	35 (4.2%)	1.000
Acute insults	*N* = 1525	*N* = 607	*N* = 918	
Alcohol	783 (51.3%)	382 (62.9%)	401 (43.7%)	< 0.0001
HBV reactivation	260 (17.0%)	75 (12.4%)	185 (20.2%)	< 0.0001
Drug-induced	126 (8.3%)	46 (7.6%)	80 (8.7%)	0.449
Acute HEV	62 (4.1%)	28 (4.6%)	34 (3.7%)	0.427
Other viral hepatitis	68 (4.5%)	24 (4.0%)	44 (4.8%)	0.527
Infection-related	30 (2.0%)	0 (0%)	30 (3.3%)	< 0.0001
Autoimmune hepatitis	48 (3.1%)	21 (3.5%)	27 (2.9%)	0.653
Other causes^b^	16 (1.0%)	3 (0.5%)	13 (1.4%)	0.122
Cryptogenic	68 (4.5%)	21 (3.5%)	47 (5.1%)	0.130
CTP score on admission	11.8 ± 1.5	11.5 ± 1.4	12.0 ± 1.6	< 0.0001
MELD score on admission	29.3 ± 7.1	28.6 ± 6.2	30.0 ± 7.5	0.001
Number of organs failure	1.68 ± 1.1	1.6 ± 1.0	1.7 ± 1.0	0.001
No organ failure	182 (11.2%)	74 (11.6%)	108 (11.0%)	
One organ failure	595 (36.7%)	263 (41.3%)	332 (33.7%)	
Two organ failure	505 (31.2%)	186 (29.2%)	319 (32.4%)	
Three organ failure	256 (15.8%)	91 (14.3%)	165 (16.8%)	
Four organ failure	70 (4.3%)	20 (3.1%)	50 (5.1%)	
Five organ failure	12 (0.7%)	3 (0.5%)	9 (0.9%)	
Six organ failure	1 (0.1%)	0 (0%)	1 (0.1%)	
Specific organs failure				
Hepatic encephalopathy	759 (46.8%)	248 (38.9%)	511 (51.9%)	< 0.0001
Coagulation	573 (35.3%)	173 (27.2%)	400 (40.7%)	< 0.0001
Renal	376 (23.2%)	106 (16.6%)	270 (27.4%)	< 0.0001
Circulatory	60 (3.7%)	16 (2.5%)	44 (4.5%)	0.004
Respiratory	224 (13.8%)	144 (22.6%)	80 (8.1%)	< 0.0001
Liver failure	1294 (79.8%)	505 (79.3%)	789 (80.2%)	0.660
Baseline laboratories				
Hemoglobin, g/dL	10.6 ± 2.2	10.6 ± 2.1	10.5 ± 2.4	0.756
WBC count, 10^9^/L	14.3 ± 9.3	14.1 ± 9.0	14.4 ± 9.5	0.646
Platelet count, 10^9^/L	145.8 ± 89.4	147.8 ± 91.0	144.5 ± 88.4	0.470
Serum sodium, mEq/L	131.0 ± 8.1	130.0 ± 7.0	132.0 ± 8.6	< 0.0001
Creatinine, mg/dL	1.6 ± 1.6	1.3 ± 1.2	1.8 ± 1.7	< 0.0001
Total bilirubin, mg/dL	21.5 ± 9.8	21.5 ± 9.9	21.6 ± 9.8	0.817
Albumin, g/dL	2.3 ± 0.7	2.2 ± 0.6	2.4 ± 0.7	< 0.0001
ALT, U/L	203.5 ± 454.5	126.0 ± 257.0	254.0 ± 541.0	< 0.0001
INR	2.6 ± 1.2	2.3 ± 0 .8	2.7 ± 1.3	< 0.0001
Length of hospital stay (days)	17.5 ± 14.1	18.0 ± 14.2	17.2 ± 14.0	0.243
Mortality rate				
28-days	637 (39.3%)	169 (26.5%)	468 (47.6%)	< 0.0001
90-days	809 (49.9%)	245 (38.5%)	564 (57.3%)	< 0.0001

*ALT* alanine aminotransferase, *CLD* chronic liver disease, *CTP* Child-Turcotte-Pugh score, *HE* hepatic encephalopathy, *HCV* hepatitis C virus, HEV; hepatitis E virus, *INR* international normalized ratio, *MELD* model for end-stage liver disease, *WBC* white blood cell count

^a^Autoimmune hepatitis, primary biliary cholangitis, Wilsons disease and cholestatic liver diseases

^b^Viral hepatitis (HAV, HDV, HEV, EBV), autoimmune hepatitis, acute Wilsons disease, infection-induced (sepsis, spontaneous bacterial peritonitis, malaria, leptospirosis) and post-surgery

### Influence of each etiology of underlying CLD

Alcohol consumption shared a greater number in cirrhosis than non-cirrhosis in terms of both CLD etiology (66.4% vs. 44.2%, *p* < 0.0001) and liver insult (63.0% vs. 38.8%, *p* < 0.0001). Regarding cryptogenic CLD cases, they were more common in non-cirrhosis than cirrhosis (10.9% vs. 5.8%, *p* < 0.0001). In addition, chronic HBV infection and reactivation was significantly more common in non-cirrhosis than cirrhosis (18.8% vs. 11.8%, *p* < 0.0001).

Baseline hematological findings (hemoglobin, white cell count (WBC), and platelet count) and total bilirubin level from both groups were similar. Non-cirrhosis CLD patients had a statistically higher level of serum sodium, creatinine, albumin, alanine aminotransferase, and INR than cirrhosis CLD patients. Baseline CTP and MELD scores were significantly higher in non-cirrhosis than cirrhosis group (12.0 ± 1.6 vs 11.5 ± 11.4, *p* < 0.0001, for CTP scores and 30.0 ± 7.5 vs 28.6 ± 6.2, *p* = 0.001, for MELD scores).

### Organ failures

Most patients had single organ failure (41.3% in cirrhosis and 33.7% in non-cirrhosis patients), multiple organ failure was more common in non-cirrhosis group (32.4% and 16.8% of patients had two and three organ failure, respectively). Liver failure was the most common type of organ failure in patients with cirrhosis (79.3%) and non-cirrhosis (80.2%). In contrast, cerebral failure was the most common extra-hepatic organ failure in those with cirrhosis (38.9%) and non-cirrhosis (51.9%). ACLF patients without cirrhosis had more proportion of cerebral, coagulation, renal and circulatory failure compared to those with cirrhosis (Table 1). The proportion of respiratory failure in patients with non-cirrhosis was less than cirrhosis (8.1% vs. 22.6%, *p* < 0.0001).

### Mortality

Only 0.8% of patients underwent liver transplantation. Overall, 28-day and 90-day mortality rates were 39.3% and 49.9%, respectively. Non-cirrhosis patients had a significantly higher mortality rate than cirrhosis patients, both at day 28 (47.6% vs. 26.5%, *p* < 0.0001) and day 90 (57.3% vs. 38.5%, *p* < 0.0001) (Table 2). However, concerning etiologies of CLD, only non-cirrhotic patients with alcoholic and cryptogenic liver disease had a significantly higher 28-day and 90-day mortality than cirrhotic patients (Table 2). In contrast, the mortality rate was not significantly different between both groups of patients in HBV, hepatitis C virus (HCV), and non-alcoholic fatty liver disease (NAFLD). In alcoholic CLD, non-cirrhosis patients had a higher 28-day (49.9% vs 23.6%, *p* < 0.001) and 90-day (58.4% vs 35.2%, *p* < 0.001) mortality rate than cirrhosis patients. Characteristics of ACLF patients with alcohol-related liver disease with or without cirrhosis were shown in Supplementary Table 1. Non-cirrhotic patients had a higher white blood cell count than cirrhotic patients. In addition, cerebral, coagulation, renal, and circulatory failure and more than two organ failures were more frequently found in ACLF patients without cirrhosis than patients with cirrhosis (Supplementary Table 1).

**Table 2:** The 28-day and 90-day mortality rate in ACLF patients with and without cirrhosis

	28-day mortality rate				90-day mortality rate			
	Cirrhosis	Non-cirrhosis	OR (95%CI)	*p*-value	Cirrhosis	Non-cirrhosis	OR (95%CI)	*p*-value
Overall	26.5% (169/637)	47.6% (468/984)	2.51 (2.02–3.12)	< 0.001	38.5% (245/637)	57.3% (564/984)	2.15 (1.75–2.63)	< 0.001
ARLD	23.6% (100/423)	49.9% (217/435)	3.22 (2.40–4.31)	< 0.001	35.2% (149/423)	58.4% (254/435)	2.58 (1.96–3.40)	< 0.001
HBV infection	39.8% (39/98)	38.6% (68/176)	0.95 (0.57–1.58)	0.90	46.9% (46/98)	48.9% (86/176)	1.08 (0.66–1.77)	0.80
HCV	10% (1/10)	37.5% (12/32)	5.40 (0.61–48.08)	0.10	30.0non% (3/10)	59.4% (19/32)	3.41 (0.74–15.68)	0/10
NASH	40.5% (15/37)	59.1% (26/44)	2.11 (0.87–5.16)	0.10	67.6% (25/37)	61.4% (27/44)	0.76 (0.31–1.91)	0.56
Cryptogenic	24.3% (9/37)	47.7% (51/107)	2.83 (1.22–6.57)	0.01	32.4% (12/37)	57.9% (62/107)	2.87 (1.31–6.31)	0.007
Other causes	19.2% (5/26)	47.2% (17/36)	3.76 (1.16–12.16)	0.02	34.6% (9/26)	63.9% (23/36)	3.34 (1.16–9.61)	0.02

*ARLD* alcohol-related liver disease, *HBV* hepatitis B virus, *HCV* hepatitis C virus, *NASH* non-alcoholic steatohepatitis

**Fig. 2:**
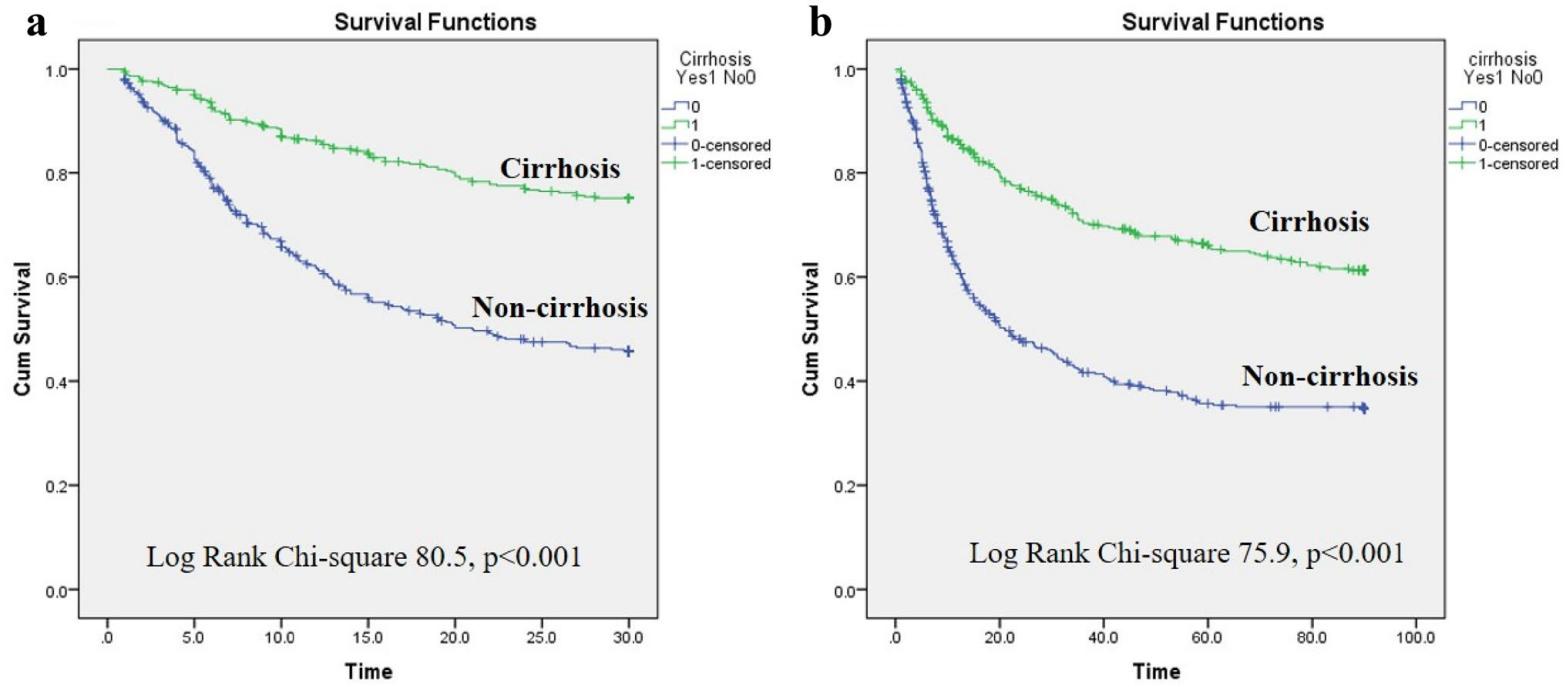
Survival according to the presence of cirrhosis or non-cirrhosis in patients with ACLF at 28 days and 90 days. **a** Survival curves at 28 days (Log Rank Chi-Square = 79.5; *p* < 0.0001). **b** Survival curves at 90 days (Log Rank Chi-Square = 73.3; *p* < 0.0001)

The survival analyses according to the presence of cirrhosis in alcoholic CLD are demonstrated in Fig. 2, which confirmed a better prognosis in cirrhosis patients rather than non-cirrhosis. Cirrhotic patients had longer survival than non-cirrhotic patients at both 28-day and 90-day analyses (25.5 vs. 18.8 days at 28-day evaluation and 65.2 vs. 41.2 days at 90-day evaluation). (Fig. 2*p*).

### Risk factors for mortality

The potential risk factors associated with the 28-day and 90-day mortality were analyzed using univariate and multivariate analysis (Tables 3 and 4). The presence of cirrhosis significantly influenced on mortality at both 28-day and 90-day, with the same trends found from crude analysis by Kaplan–Meier methods. CLD patients with cirrhosis had a greater chance to survive compared to CLD patients without cirrhosis, as demonstrated by the hazard ratio at both 28-days and 90-days were 0.48 (95% CI 0.36–0.63, *p* < 0.0001) and 0.56 (95% CI 0.43–0.72, *p* < 0.0001), respectively. Among the pre-ACLF parameters, age was a variable associated with a poorer prognosis, while acute injury from alcohol was independently associated with lower 28-day mortality. Sex and etiologies of CLD were not independently associated with 28-day and 90-day mortality. Regarding the post-ACLF parameters, defined as parameters presented at the time of ACLF diagnosis, we found that total WBC count, MELD scores, number of organ failures, and baseline hepatic encephalopathy were significantly associated with more significant mortalities at both 28-day and 90-day.

**Table 3:** Univariate and multivariate analysis of factors associated with the 28-day mortality in patients with acute-on-chronic liver failure

Variables	Univariate analysis			Multivariate analysis		
	*p*-value	Hazard ratio	95% CI	*p*-value	Hazard ratio	95% CI
Presence of cirrhosis	< 0.0001	0.56	0.46–0.69	< 0.001	0.48	0.36–0.63
Age	0.02	1.01	1.00–1.02	0.09	1.01	1.00–1.02
Male sex	0.88	0.98	0.72–1.34			
Acute insult (Alcohol vs. non-alcohol)	0.02	0.79	0.65–0.97	0.04	0.75	0.56–0.99
CLD etiologies	0.36	1.01	0.99–1.03			
Hemoglobin	0.10	0.96	0.92–1.01			
WBC count	0.002	1.01	1.01–1.02	< 0.001	1.03	1.02–1.05
Platelet count	0.21	1.00	0.99–1.00			
Serum sodium	0.40	1.01	0.99–1.02			
Creatinine	0.21	1.04	0.98–1.10			
Total bilirubin	0.44	1.00	0.99–1.02			
Albumin	0.48	0.94	0.79–1.12			
ALT	0.05	1.00	1.000–1.000			
INR	0.005	1.15	1.04–1.26	0.99	1.00	0.87–1.16
CTP scores	0.19	1.05	0.98–1.13			
MELD scores	< 0.0001	1.07	1.04–1.09	< 0.001	1.11	1.08–1.14
Number of organ failure	0.005	1.20	1.06–1.36	0.04	1.21	1.01–1.44
Presence of ascites	0.08	1.41	0.96–2.08			
Presence of HE^a^	< 0.0001	2.03	1.64–2.52	< 0.001	2.82	2.16–3.67

*ALT* alanine aminotransferase, *CLD* chronic liver disease, *CTP* Child-Turcotte-Pugh score, *HE* hepatic encephalopathy, *INR* international normalized ratio, *MELD* model for end-stage liver disease, *WBC* white blood cell count

^a^Hepatic encephalopathy by any severity

**Table 4:** Univariate and multivariate analysis of factors associated with the 90-day mortality in patients with acute-on-chronic liver failure

Variables	Univariate analysis			Multivariate analysis		
	*p*-value	Hazard ratio	95% CI	*p*-value	Hazard ratio	95% CI
Presence of cirrhosis	< 0.0001	0.61	0.51–0.73	< 0.001	0.56	0.43–0.72
Age	0.001	1.01	1.01–1.02	0.05	1.01	1.00–1.02
Male sex	0.97	1.01	0.77–1.32			
Acute insult (Alcohol vs. non-alcohol)	0.03	0.81	0.66–0.98	0.06	0.76	0.58–1.01
CLD etiologies	0.39	1.01	0.99–1.03			
Hemoglobin	0.04	0.96	0.92–0.99	0. 14	0.96	0.90–1.02
WBC count	0.001	1.01	1.01–1.02	0.001	1.03	1.01–1.05
Platelet count	0.04	0.999	0.99–1.00	0.001	0.998	0.996–0.999
Serum sodium	0.99	1.00	0.99–1.01			
Creatinine	0.36	1.03	0.97–1.08			
Total bilirubin	0.03	1.01	1.00–1.02	0.02	1.02	1.00–1.03
Albumin	0.64	0.96	0.82–1.13			
ALT	0.08	1.00	1.000–1.000			
INR	0.005	1.14	1.04–1.24	0.85	1.01	0.88–1.17
CTP scores	0.09	1.06	0.99–1.13			
MELD scores	< 0.0001	1.05	1.03–1.08	< 0.001	1.07	1.04–1.10
Number of organ failure	0.001	1.21	1.08–1.36	0.002	1.32	1.11–1.57
Presence of ascites	0.07	1.38	0.98–1.94			
Presence of HE^a^	< 0.0001	1.66	1.38–2.00	< 0.001	1.96	1.52–2.52

*ALT* alanine aminotransferase, *CLD* chronic liver disease, *CTP* Child-Turcotte-Pugh score, *HE* hepatic encephalopathy, *INR* international normalized ratio, *MELD* model for end-stage liver disease, *WBC* white blood cell count

^a^Hepatic encephalopathy by any severity

## Discussion

This study analyzed the information from the AARC registry, the largest ACLF patients database in the Asia–Pacific region. In comparison with the previous information published by the APASL in 2014 [4], the overall 28-day mortality rate decreased from over 50% in the previous analysis to 39.3% in the current study. This might reflect an improvement in the quality of care for ACLF patients. In terms of the CLD etiologies and precipitating causes, the trend of prevalence in each factor was similar to the study in 2014, but these differed from the first evaluation in 2009. HBV infection was the most common cause of CLD, and HBV reactivation was the most common precipitating factor in 2009. HBV prevalence was predominately found among Asian countries, whereas alcohol-related complications were the major contributing factor at both acute and chronic liver injury in Western countries [7]. The current analysis showed that alcohol consumption surpassed others to become the major cause of CLD and liver injury, while HBV-related complications dropped to second rank (Table 1). It should be noted that the prevalence of NAFLD from our study was much increased. Furthermore, the incidence of NAFLD might be under-detected because the burnt-out NASH might be misdiagnosed as cryptogenic [4]. Based on these situations, it might be predicted that according to the westernization of lifestyle of the Asian people and the significant changes in dietary habits, the incidence of CLD and ACLF would exponentially increase, and NAFLD might be one of the contributing factors for these epidemiological shifts in CLD etiologies. Changing trends were not only discovered in the Asia–Pacific region but also observed globally [8, 9].

Several variables that influence ACLF prognosis has been discovered; however, there were conflicting associations between each parameter to the outcome. Apart from the presence of cirrhosis, aging and acute insult from alcohol were the pre-ACLF factors that influence ACLF mortality, while other parameters, including sex and types of CLD etiologies, which were found potentially related with prognosis by univariate analysis. These parameters were found insignificant to mortality after being adjusted with the presence of cirrhosis by multivariate analysis. Shi et al. [10] showed that cirrhotic patients who were injured by non-liver insults, such as bacterial infection, were related with a worse prognosis than liver-related insults, such as alcohol drinking. Shalimar et al. [11] found alcohol and cryptogenic liver insults were independent risk factors to mortality. They also found that HEV CLD patients had a significantly better prognosis than other CLD patients. In concordance with the results of most literature, the higher level of post-ACLF parameters in our study including WBC count, MELD scores, the number of organ failures and hepatic encephalopathy were associated with poorer prognosis. The presence of cirrhosis was an independent factor of mortality. However, these parameters were not observed as significant effects by prior literatures and systematic reviews [11–20]. Paradoxical impacts from each variable might be explained by the small sample size and the heterogeneity of the study protocol and ACLF definition among different studies [12].

The 28-day and 90-day mortality rates in cirrhosis patients were significantly lower than in non-cirrhosis patients. Both univariate and multivariate analyses confirmed that the presence of cirrhosis significantly impacted the prognosis among ACLF patients. Cirrhosis ACLF patients had about a 50% greater chance to survive at both 28-days and 90-days in comparison to those without cirrhosis. To compare our results with other literature, only a few studies had evaluated the effects of cirrhosis on mortality. Three studies evaluated CLD patients from any cause. They did not find noteworthy differences between the presence of cirrhosis and short-term (28-days) and long-term (after 28-days) mortalities [17, 21, 22]. Four literatures evaluated the prognostic effects among HBV-related ACLF patients. Every study performed multivariate analysis considering the presence of cirrhosis as one of the prognostic parameters. Interestingly, none of these studies evaluated short-term mortality. Three studies did not find any critical impacts from the presence of cirrhosis to long-term mortality (equal or more than three months) [15, 16, 19]. Another study found that liver cirrhosis was an independent risk factor for 3-month death [14].

Although most literature did not find significant effects of the presence of cirrhosis on prognosis, which contrasted results from our study, we should take into account the fact that these results were not the primary objective of their studies, so the number of the study population might be too small to discriminate the effect of cirrhosis on mortality. The significant impact of the presence of cirrhosis was consistently observed from every evaluation in our study, in which the study protocol was specifically generated. Moreover, our database was relatively large and enrolled diverse participants from various countries and ethnicities. This was the strength of our study, reassuring that the presence of cirrhosis influenced ACLF prognosis particularly in patients who had alcohol-related liver disease.

Systemic inflammatory response after liver injury is a major contributing mechanism in the pathogenesis of ACLF. There are two types of triggers that are able to stimulate inflammatory responses. Exogenous triggers, such as bacterial infection stimulate different types of inflammatory cells and cytokine pathways that give rise to inflammation. Endogenous triggers originating from necrotic hepatocytes and products from extracellular matrix breakdown induce inflammation to promote tissue restoration. Two types of inducers act synergistically in response to the liver injury to stimulate inflammatory cascade and repair mechanisms; however, excessive inflammation could adversely bring about organ failure and death [23]. Besides the inappropriate immune response after liver injury, gut dysbiosis and bacterial translocation found in many CLD situations also play a role in the development of ACLF. They additionally activate inflammatory responses and interact with the precipitated-inflammatory cascade. As a result, the inflammatory response that was already dysregulated would be more heavily damaged [5, 6, 24].

Two possible reasons might explain why patients with non-cirrhosis had a worse prognosis than patients with cirrhosis after ACLF development in alcohol-related liver disease. First, non-cirrhosis had a more inappropriate and exaggerated immune response than cirrhosis. Although ACLF patients did not have cirrhosis, they still developed systemic inflammation, which repetitively or continuously provoked an immune response. The study showed that white blood cell count was higher in alcoholic CLD patients with non-cirrhosis, demonstrating a higher degree of systemic inflammatory response. Additionally, patients with non-cirrhosis had more cerebral, coagulation, renal, and circulatory failure than patients with cirrhosis. Systemic inflammation is a distinct characteristic of ACLF, and it is significantly more prevalent in alcoholic liver disease-ACLF [25–27]. An alteration in intestinal microbiomes, which is the supported mechanism of ACLF, was also observed in patients with non-cirrhosis CLD and alcohol-related liver disease by many recent literatures [28–30]. Increasing evidence of an imbalance of gut microbiota and bacterial translocation was frequently found in alcoholic CLD [31–33]. These might be the possible explanations for why non-cirrhosis had a poorer prognosis in patients with alcoholic CLD. However, we have no data on the inflammatory parameters in both groups of ACLF, and this hypothesis needs further studies to confirm. Second, the limitation of ACLF definition, patients with decompensated cirrhosis who were likely to have poorer outcomes, had been excluded. These might lead to the lower mortality in ACLF patients who had the background of liver cirrhosis.

Our study demonstrated that although non-cirrhosis CLD patients had a lower chance of liver-related complications, they were equally or at higher risk of poor outcome in cases with complications than patients with cirrhosis. While most physicians focused their concerns on cirrhosis patients, we recommended increasing the level of attention to CLD patients particularly alcoholic CLD. At ACLF presentation, non-cirrhosis patients should be concerned that they might have serious systemic inflammation. We encourage including the presence of cirrhosis with the type and number of organ failures at an initial evaluation for severity grading.

Our study had some limitations. First, there was marked heterogeneity in treatment protocols between each institution. We did not include treatment effects, particularly liver transplantation, in prognosis assessment. In addition, only a small number of patients (0.8%, *n* = 13) underwent liver transplantation, resulting in difficulty in analyzing its relation with prognostic outcomes. Second, we did not collect and compare inflammatory biomarkers between cirrhosis and non-cirrhosis ACLF patients to confirm our hypothesis. Hence, we suggest collecting inflammatory biomarkers for future research, which might help establish the hypothesis of the exaggerated inflammation and identify correlations with prognosis.

## Conclusion

Baseline cirrhosis in ACLF patients should be thoroughly evaluated. Non-cirrhotic patients with alcoholic CLD who developed ACLF were associated with higher 28-day and 90-day mortality rates, which might indicate the state of overwhelming inflammation and need closed monitoring, not lesser than patients with cirrhosis.

## Supplementary Information

Below is the link to the electronic supplementary material.

Supplementary file1 (DOCX 16 kb)

## Declarations

**Collaborators** The following members of the DUCA group were collaborators in this study: Shiv Kumar Sarin, Ashok Choudhury, Manoj K. Sharma1, Rakhi Maiwall1 (Department of Hepatology, Institute of Liver and Biliary Sciences, New Delhi 110070, India); Mamun Al Mahtab, Salimur Rahman (Department of Hepatology, Bangabandhu Sheikh Mujib Medical University, Dhaka, Bangladesh); Sanjiv Saigal, Neeraj Saraf, A. S. Soin (Department of Hepatology, Medanta The Medicity, Gurgaon, India); Harshad Devarbhavi (Department of Internal Medicine, Hallym University College of Medicine, Seoul, South Korea); Dong Joon Kim (Department of Internal Medicine, Hallym University College of Medicine, Seoul, South Korea); R. K. Dhiman, Ajay Duseja, Sunil Taneja (Department of Hepatology, PGIMER, Chandigarh, India); C. E. Eapen, Ashish Goe (Department of Hepatology, CMC, Vellore, India); Q. Ning (Institute and Department of Infectious Disease, Tongji Hospital, Tongji Medical College, Huazhong University of Science and Technology, Wuhan, China); Tao Chen (Translational Hepatology Institute Capital Medical University, Beijing You’an Hospital, Beijing, China); Ke Ma (Institute and Department of Infectious Disease, Tongji Hospital, Tongji Medical College, Huazhong University of Science and Technology, Wuhan, China); Z. Duan, Chen Yu (Translational Hepatology Institute Capital Medical University, Beijing You’an Hospital, Beijing, China); Sombat Treeprasertsuk (Department of Medicine, Chulalongkorn University, Bangkok, Thailand); S. S. Hamid, Amna S. Butt, Wasim Jafri (Department of Medicine, Aga Khan University Hospital, Karachi, Pakistan): Akash Shukla (Department of Gastroenterology, Lokmanya Tilak Municipal General Hospital and Lokmanya Tilak Municipal Medical College, Sion, Mumbai, India); Vivek Saraswat (Department of Gastroenterology, SGPGIMS, Lucknow, India); Soek Siam Tan (Department of Medicine, Hospital Selayang, Bata Caves, Selangor, Malaysia); Ajit Sood, Vandana Midha, Omesh Goyal (Department of Gastroenterology, DMC, Ludhiana, India), Hasmik Ghazinyan (Department of Hepatology, Nork Clinical Hospital of Infectious Disease, Yerevan, Armenia), Anil Arora (Department of Gastroenterology and Hepatology, Sir Ganga Ram Hospital and GRIPMER, New Delhi, Delhi, India), Jinhua Hu (Department of Medicine, 302 Millitary Hospital, Beijing, China); Manoj Sahu (Department of Gastroenterology and Hepatology Sciences, IMS & SUM Hospital, Bhubaneswar, Odisha, India); P. N. Rao (Asian Institute of Gastroenterology, Hyderabad, India); Guan H. Lee, Seng G. Lim (Division of Gastroenterology and Hepatology, Department of Medicine, National University Health System, Singapore, Singapore); Laurentius A. Lesmana, Cosmas Rinaldi Lesmana (Digestive Disease and GI Oncology Centre, Medistra Hospital, Jakarta, Indonesia); Samir Shah (Department of Hepatology, Global Hospitals, Mumbai, India); V. G. Mohan Prasad (Department of Gastroenterology, VGM Hospital, Coimbatore, India); Diana A. Payawal (Fatima University Medical Center Manila, Manila, Philippines); Zaigham Abbas (Department of Medicine, Ziauddin University Hospital, Karachi, Pakistan); A. Kadir Dokmeci (Department of Medicine, Ankara University School of Medicine, Ankara, Turkey); Jose D. Sollano, Gian Carpio (Department of Medicine, University of Santo Tomas, Manila, Philippines); Ananta Shresta (Department of Hepatology, Foundation Nepal Sitapaila Height, Kathmandu, Nepal); G. K. Lau (Department of Medicine, Humanity and Health Medical Group, New Kowloon, Hong Kong, China); ·Md. Fazal Karim (Department of Hepatology, Sir Salimullah Medical College, Dhaka, Bangladesh); Gamal Shiha (Egyptian Liver Research Institute And Hospital, Cairo, Egypt); Rino Gani, Kemal Fariz Kalista (Division of Hepatobiliary, Department of Internal Medicine, Faculty of Medicine, Cipto Mangunkusumo Hospital, Universitas Indonesia, Jakarta, Indonesia); Man-Fung Yuen (Department of Medicine, Queen Mary Hospital Hong Kong, The University of Hong Kong, Hong Kong, China); Seema Alam, Rajeev Khanna, Vikrant Sood, Bikrant Bihari Lal (Department of Pediatric Hepatology, Institute of Liver and Biliary Sciences, New Delhi, Delhi, India; Viniyendra Pamecha (Department of Hepatobilliary Pancreatic Surgery and Liver Transplant, Institute of Liver and Biliary Sciences, New Delhi, Delhi, India); Ankur Jinda (Department of Medicine, Aga Khan University Hospital, Karachi, Pakistan); V. Rajan, Vinod Arora (Department of Hepatology, Institute of Liver and Biliary Sciences, New Delhi 110070, India); Osamu Yokosuka (Professor Emeritus, Chiba University, Chiba, Japan); Madunil A. Niriella (Department of Medicine, University of Kelaniya, Ragama, Sri Lanka); Hai Li (Department of Gastroenterology, Ren Ji Hospital, School of Medicine, Shanghai Jiao Tong University, Shanghai, China); Xiaolong Qi (CHESS Frontier Center, The First Hospital of Lanzhou University, Lanzhou University, Lanzhou, China); Atsushi Tanaka (Department of Medicine, Tokyo University School of Medicine, Tokyo, Japan); Satoshi Mochida (Department of Gastroenterology and Hepatology, Faculty of Medicine, Saitama Medical University, Saitama, Japan); Dominic Ray Chaudhuri, Ed Gane (New Zealand Liver Transplant Unit, Auckland Hospital, Auckland, New Zealand); Khin Maung Win (Yangon GI and Liver Centre, Pabedan, Yangon, Myanmar); Wei Ting Chen (Division of Hepatology, Department of Gastroenterology and Hepatology, Chang Gung Medical Foundation, Linkou Chang Gung Memorial Hospital, Taoyuan, Taiwan); Mohd. Rela (Department of Liver Transplant Surgery, Dr. Rela Institute and Medical Centre, Chennai, India); Dharmesh Kapoor (Department of Hepatology, Global Hospitals, Mumbai, India); Amit Rastogi (Department of Hepatology, Medanta The Medicity, Gurgaon, India); Pratibha Kale (Department of Microbiology, Institute of Liver and Biliary Sciences, New Delhi, Delhi, India); Archana Rastogi, Chhagan Bihari Sharma (Department of Pathology, Institute of Liver and Biliary Sciences, New Delhi, Delhi, India); Meenu Bajpai (Department of Immunohematology and Transfusion Medicine, Institute of Liver and Biliary Sciences, New Delhi, Delhi, India); Virender Singh, Madhumita Premkumar (Department of Hepatology, PGIMER, Chandigarh, India); Sudhir Sudhir · A. Olithselvan (Division of Liver Transplantation and Hepatology, Manipal Hospitals, Bangalore, India); Cyriac Abby Philips (The Liver Unit, Cochin Gastroenterology Group, Ernakulam Medical Centre, Kochi, India); Anshu Srivastava, Surender K. Yachha (Department of Pediatric Gastroenterology, SGPGIMS, Lucknow, India); Zeeshan Ahmad Wani (Noora Hospital in Srinagar, Shrinagar, India); B. R. Thapa (Department of Gastroenterology and Pediatric Gastroenterology, PGIMER, Chandigarh, India); Anoop Saraya, Shalimar (Department of Gastroenterology and Human Nutrition, AIIMS, New Delhi, India); Ashish Kumar (Department of Gastroenterology and Hepatology, Sir Ganga Ram Hospital and GRIPMER, New Delhi, Delhi, India); Manav Wadhawan (Department of Gastroenterology, Hepatology and Liver Transplant, B L K Hospital, New Delhi, India); Subash Gupta (Centre for Liver and Biliary Science, Max Hospital, New Delhi, India); Kaushal Madan (Department of Gastroenterology, Hepatology and Liver Transplant, Max Hospital, New Delhi, India); Puja Sakhuja (Department of Pathology, GB Pant Hospital, New Delhi, India); Vivek Vij (Department of Liver Transplant and Hepatobilliary Surgery, Fortis Hospital, New Delhi, India);Barjesh C. Sharma (Department of Gastroenterology, GB Pant Hospital, New Delhi, India); Hitendra Garg, Vishal Garg (Department of Gastroenterology, Hepatology and Liver Transplant, Apollo Hospital, New Delhi, India); Chetan Kalal (Department of Hepatology, Sir H N Reliance Hospital and Research Centre, Mumbai, India); Lovkesh Anand (Department of Gastroenterology and Hepatology, Narayana Hospital, Gurugram, India); Tanmay Vyas (Department of Hepatology, Parimal Multi-Speciality Hospital, Ahmedabad, India); Rajan P. Mathur (Department of Nephrology, Institute of Liver and Biliary Sciences, New Delhi, India); Guresh Kumar, Priyanka Jain, Samba Siva Rao Pasupuleti (Department of Statistics and Clinical Research, Institute of Liver and Biliary Sciences, New Delhi, India); Yogesh K. Chawla (Department of Hepatology and Gastroenterology, Kalinga Institute of Med Sciences, KIIT University, Bhubaneswar, India); Abhijit Chowdhury (Department of Hepatology, Institute of Post Graduate Medical Education and Research, Kolkata, India); Shahinul Alam (Department of Hepatology, Bangabandhu Sheikh Mujib Medical University, Dhaka, Bangladesh); Do Seon Song, Jin Mo Yang (Department of Internal Medicine, College of Medicine, The Catholic University of Korea, Seoul, South Korea); Eileen L. Yoon (Department Of Internal Medicine, Inje University College of Medicine, Busan, South Korea).
