# Supplementary material for: Impact of compensated cirrhosis on survival in patients with acute-on-chronic liver failure
Source: Hepatol Int. 2021 Nov 25;16(1):171–82. doi: 10.1007/s12072-021-10266-8 (PMC8844167; doi:10.1007/s12072-021-10266-8)
Supplement: Supplementary file 1 — Supplementary file1 (DOCX 16 kb) [file 12072_2021_10266_MOESM1_ESM.docx]

**Supplementary Table 1 Characteristics of acute-on-chronic liver failure patients with alcohol-related liver disease**

| **Characteristics** | **Overall**  **(n =858)** | **Cirrhosis**  **(n =423)** | **Non-cirrhosis**  **(n =435)** | ***P*-value** |
| --- | --- | --- | --- | --- |
| **Age (years)** | 42.57+9.41 | 41.96+8.96 | 43.16+9.80 | 0.06 |
| **Male, n (%)** | 843 (98.3%) | 415 (98.1%) | 428 (98.4%) | 0.80 |
| **Acute insults**   - Alcohol - HBV reactivation - Drug-induced - Acute HEV - Other viral hepatitis - Infection-related - Autoimmune hepatitis - Other causes^‡^ - Cryptogenic | 745 (86.83%)  7 (0.82%)  36 (4.20%)  21 (2.45%)  23 (2.68%)  7 (0.82%)  15 (1.75%)  2 (0.23%)  2 (0.23%) | 371 (87.71%)  5 (1.18%)  13 (3.07%)  15 (3.55%)  10 (2.33%)  0 (0%)  8 (1.89%)  1 (0.24%)  0 (0%) | 374 (85.98%)  2 (0.46%)  23 (5.29%)  6 (1.38%)  13 (2.99%)  7 (1.61%)  7 (1.61%)  1 (0.23%)  2 (0.46%) | 0.48  0.28  0.13  0.05  0.67  0.02  0.8-  1.00  0.50 |
| **CTP score on admission (median, IQR)** | 12 (6-15) | 12 (6-15) | 12 (8-15) | <0.001 |
| **MELD score on admission** | 29.66+7.01 | 28.59+6.14 | 30.70+7.63 | <0.001 |
| **Number of organs failure** |  |  |  |  |
| - No organ failure - One organ failure - Two organ failure | 99 (11.54%)  298 (34.73%)  261 (30.42%) | 54 (12.77%)  174 (41.13%)  123 (29.08%) | 45 (10.34%)  124 (28.51%)  138 (31.72%) | 0.29  <0.001  0.42 |
| - More than two organ failure | 200 (23.3%) | 72 (17%) | 128 (29.4%) | <0.001 |
| **Specific organs failure** |  |  |  |  |
| - Hepatic Encephalopathy | 99 (11.54%) | 30 (7.09%) | 69 (15.86%) | <0.001 |
| - Coagulation | 281 (32.75%) | 106 (25.06%) | 175 (40.23%) | <0.001 |
| - Renal | 237 (27.62%) | 79 (18.68%) | 158 (36.3%) | <0.001 |
| - Circulatory | 35 (4.08%) | 9 (2.13%) | 26 (5.98%) | 0.003 |
| - Respiratory | 151 (17.60%) | 104 (24.59%) | 47 (10.80%) | <0.001 |
| - Liver failure | 676 (78.79%) | 326 (77.07%) | 350 (80.46%) | 0.24 |
| **Baseline laboratories** |  |  |  |  |
| - Hemoglobin, g/dL | 9.99+2.01 | 10.22+1.94 | 9.76+2.05 | 0.001 |
| - WBC count, 10^9^/L | 17.64+10.49 | 16.34+9.81 | 18.90+10.97 | <0.001 |
| - Platelet count, 10^9^/L | 150.66+91.75 | 152.67+91.55 | 148.70+92.00 | 0.53 |
| - Serum sodium, mEq/L | 129.73+8.29 | 129.04+7.08 | 130.39+9.09 | 0.02 |
| - Creatinine, mg/dL | 1.74+1.69 | 1.40+1.41 | 2.09+1.86 | <0.001 |
| - Total bilirubin, mg/dL | 21.39+9.80 | 20.77+9.77 | 21.99+9.80 | 0.07 |
| - Albumin, g/dL | 2.12+0.57 | 2.11+0.52 | 2.12+0.61 | 0.78 |
| - ALT, U/L | 66.37+103.88 | 66.83+106.72 | 65.92+101.12 | 0.90 |
| - INR | 2.44+0.91 | 2.31+0.78 | 2.57+1.00 | <0.001 |
| **Length of hospital stay (days)** | 14 (1-110) | 15 (1-110) | 13 (1-75) | 0.02 |
